# Endoscopic management of greater trochanteric pain syndrome (GTPS): a comprehensive systematic review

**DOI:** 10.1007/s00590-024-04019-0

**Published:** 2024-06-11

**Authors:** Riccardo Giai Via, Ahmed Elzeiny, Marco Bufalo, Alessandro Massè, Matteo Giachino

**Affiliations:** 1 Centro Traumatologico Ortopedico (CTO), Department of Orthopaedic Surgery, Department of Orthopaedic and Traumatology, University of Turin, Via Gianfranco Zuretti 29, 10126 Turin, Italy; 2 Department of Orthopaedics and Traumatology, Faculty of Medicine, Kafr El Sheikh University, Kafr El Sheikh, Egypt

**Keywords:** GTPS; Greater Trochanteric Pain Syndrome, Endoscopic Surgery, Chronic Hip Pain, Abductor Tendinopathy, Hip Arthroscopy

## Abstract

**Background:**

Greater trochanteric pain syndrome (GTPS) presents challenges in clinical management due to its chronic nature and uncertain etiology. Historically attributed to greater trochanteric bursitis, current understanding implicates abductor tendinopathy as the primary cause. Diagnosis usually involves a clinical examination and additional tests such as imaging and provocative testing. Surgical intervention may be considered for cases refractory to conservative therapy, with endoscopic techniques gaining ground over open procedures.

**Materials and methods:**

A systematic review was conducted adhering to the PRISMA guidelines. Relevant studies were searched in four databases: Pubmed, Scopus, Embase, and Medline. The selected articles were evaluated according to the criteria of levels of evidence (LoE). The Coleman methodology score (mCMS) was used to analyze the retrospective studies. This systematic review was registered in the International Prospective Registry of Systematic Reviews.

**Results:**

Surgical success rates ranged from 70.6–100%, significantly improving pain and function. Complications were generally mild, mainly hematomas and seromas, while recurrence rates were low. However, limitations such as the retrospective design and the absence of control groups warrant cautious interpretation of the results.

**Conclusions:**

Endoscopic surgery emerges as a promising option for refractory GTPS, offering effective symptom relief and functional improvement. Despite limitations, these results suggest a favorable risk–benefit profile for endoscopic procedures. Further research is needed, particularly prospective randomized trials, to confirm these findings and optimize surgical techniques to improve patient outcomes.

**Supplementary Information:**

The online version contains supplementary material available at 10.1007/s00590-024-04019-0.

## Introduction

Greater trochanteric pain syndrome (GTPS) is characterized by chronic lateral hip pain, yet its exact cause remains unclear. GTPS has an incidence rate of 1.8 individuals per 1000 per year and typically occurs between the fourth and sixth decades of life. Women have significantly higher prevalence rates (60–80%) [1, 2], probably due to such as hormonal alterations especially during menopause; anatomical causes can also predispose such as low cervical-diaphyseal angles, prosthetic replacement surgery with increased offset, or even mechanical causes such as occurs in runners [2, 3].

Several pathophysiological mechanisms have been proposed, including inflammation of the bursa due to friction of the iliotibial band (ITB) or repetitive microtraumas of the abductor muscles resulting in gluteal tendinopathy. Although in the past it was mainly attributed to greater trochanteric bursitis, contemporary literature indicates that abductor tendinopathy is the predominant cause of GTPS, affecting 18 to 50% of patients [4, 5]. Thus, this syndrome includes tendinopathies of the medius and gluteus minimus or trochanteric bursitis that may be isolated or secondary to external hip snapping [6, 7].

Symptoms of GTPS include tenderness of the lateral hip on palpation, discomfort when lying on the affected side, pain during weight-bearing activities such as walking, climbing stairs, standing, and running, as well as discomfort during prolonged sitting, resistance to abduction, and pain when sitting with crossed legs or weakness of the hip abductors. Since gluteal tendinopathy is a well-known cause of GTPS patients often present a positive Trendelenburg sign [8].

The diagnosis of GTPS is clinical and is featured by positive Little’s test characterized by pain on deep palpation at the level of the greater trochanter (GT), this sign is considered almost pathognomonic of this clinical condition [9]. Several conditions should be included in the differential diagnosis with this pathology, such as lower limb dysmetria, osteoarthritis, femoroacetabular impingement (FAI), tendon degeneration, compression of lumbar nerve roots [10, 11]. Pelvis and hip X-rays are often the initial investigation in primary care to rule out common differentials such as hip osteoarthritis or FAI and they may detect some calcifications in the tendon insertion area near the GT [12]. Ultrasound and MRI are second-tier imaging techniques for GTPS, which often reveal evidence of gluteal tendinopathy or musculotendinous tears [13].

First line treatment for GTPS is conservative with rest, physical therapy, lifestyle modifications, weight loss, and anti-inflammatory drugs. Most cases resolve with conservative treatments, but some less responsive patients may benefit from infiltrative treatment with corticosteroids or Platelet Rich Plasma (PRP) targeting the bursa at the level of the GT [14]. Surgical treatment can be proposed as a solution in cases that do not respond to conservative therapy, in recalcitrant cases, or in cases of recurrence of symptoms. In the past, open surgery of bursectomy and Z- or N-plasty of the fascia lata was often performed, but in the present day, with the improvement of arthroscopic techniques at the hip, endoscopic surgery is increasingly becoming a viable surgical option [15].

This systematic review aims to demonstrate that endoscopic surgery is a feasible alternative for treating patients with GTPS that do not respond to conservative treatments, compared to open surgery which involves greater invasiveness and higher complication rates.

Therefore, this systematic review’s main purpose is to evaluate the indications, clinical outcomes, complications, and revision rates associated with endoscopic surgery for GTPS.

## Material and methods

This systematic review is in accordance with the Preferred Reporting Items for Systematic Reviews and Meta-Analyses (PRISMA) guidelines [16]. Three authors (RGV, AE, and MB) independently conducted the literature search and evaluated the studies to reduce errors to a minimum. Uncertainties were solved by consulting a fourth author (MG).

### Inclusion and exclusion criteria

The reviewed articles, published between 2004 and February 2024, had to meet certain requirements: they had to be about patients with GTPS treated with endoscopic technique, be written in English, focus on human subjects, and have a follow-up period of at least 6 weeks on average. Randomized controlled trials (RCTs), prospective and retrospective studies with levels of evidence (LoE) between 1 and 4 were included [17]. Biochemical and in vitro studies, case reports, editorials, book chapters, technical reports, preclinical studies, review articles, and studies with LoE 5 were excluded to ensure a higher quality study.

### Search strategy and study screening

**Fig. 1:**
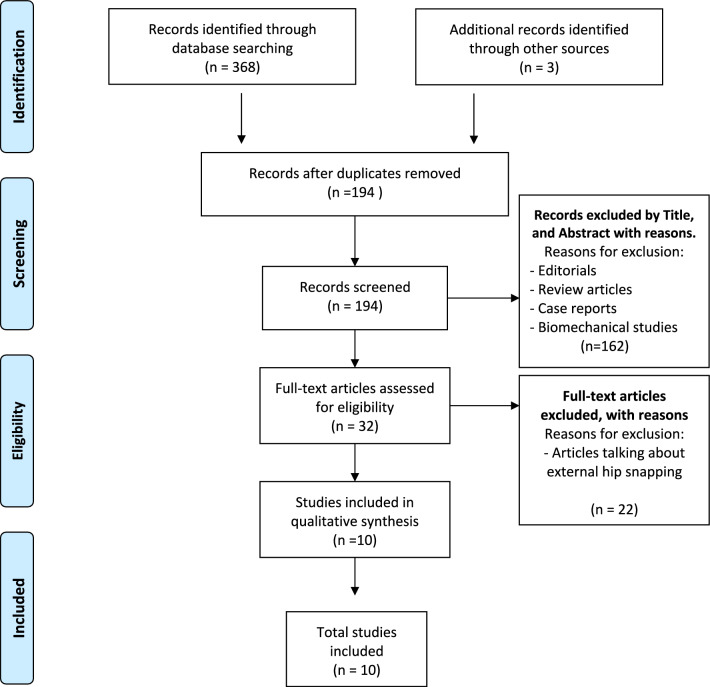
PRISMA flow diagram

A thorough and systematic literature search was conducted in four databases (PubMed, Scopus, Embase and Medline) employing the following MeSH terms: ((greater trochanter* pain syndrome) OR (GTPS)) AND ((endoscop*) OR (arthroscop*)). The research included studies published between 2004 and February 2024. The research included studies published between 2000 and February 2024. After removal of duplicates, 194 studies were included. After a revision of the title and abstract of these studies, 162 were excluded, resulting in 32 eligible studies. After full-text evaluation, 10 studies met the criteria of eligibility for qualitative analysis. The PRISMA chart is shown in Fig. 1.

### Methodological quality assessment

**Fig. 2:**
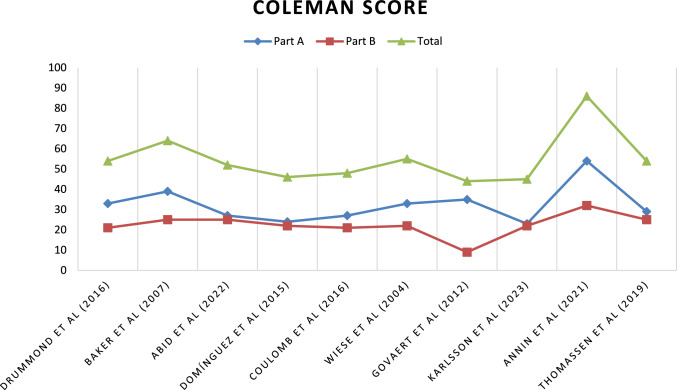
The Coleman methodology score (mCMS), modified by Ramponi et al. [18]

All included articles were evaluated according to the Oxford Centre for Evidence-Based Medicine 2011 Levels of Evidence (LoE), which ranges from 1–5. The Coleman Methodology Score (mCMS), modified by Ramponi et al., was employed for retrospective studies [18, 19] as shown in Fig. 2. This tool was used by two authors (RGV, AE), with a third author (MG) consulted for solving uncertainties. This systematic review was registered in the International Registry of Systematic Reviews (PROSPERO), CRD42024522616, in March 2024 [20].

### Data extraction

Data extracted from the included articles were meticulously registered in Excel spreadsheets by three independent authors (RGV, AE and MB) and then merged. This included details such as the author and year of publication, study design, patient sample size, mean age, mean BMI, mean period symptoms, mean follow-up time, surgical technique performed, rehabilitation protocols used, complication rates, recurrence rates, revision rates, pre and post operative subjective scores such as mHSS (modified Harris Hip Score),VAS (Visual Analogue Scale), NRS (Numerical Rating Scale), HAGOS (Copenhagen Hip and Groin Outcome Score), iHOT12 (International Hip Outcome Tool), NAHS (Non Arthritic Hip Score). This made organized data extraction and analysis easier, providing a comprehensive understanding of the study results.

### Statistical analysis

R software (2022 version 4.1.3), developed by the R Core Team in Vienna, Austria, was used for statistical analysis. Descriptive statistical methods were used for the data collected from the included studies. Mean values were calculated for continuous variables, while variability was assessed through measures such as standard deviation (SD) or range (minimum–maximum). Absolute numbers and frequency distributions were determined for categorical variables.

## Results

### Demographics of the studies

A total of ten studies were included for this systematic review [13, 21–29], among which three prospective studies and seven retrospective series analyzed the clinical outcomes of patients undergoing endoscopic treatment for greater trochanteric pain syndrome between 2004 and February 2024.

These studies included 498 patients (530 hips), of whom only 74 were male. The mean follow-up ranged from 6 weeks–44.4 months. The mean age of patients at the time of surgery ranged from 40 to 65 years in the included studies. Data on the time interval from symptom onset to surgery were reported in only three studies [22, 24, 27], while BMI information was available in only two studies [24, 28]. (Table 1**)** presents the study category, mean follow-up duration, study size, BMI, and symptomatic period.

**Table 1:** Main demographic characteristics of patients collected in studies included in the systematic review

Authors (year)	Study design (LOE)	No of patients (hips)	Age years (range)	m/f	BMI kg/m2 (range)	FU months (range)	symptomatic period years (range)
Drummond et al. [13]	Retrospective (III)	49 (57)	65 (26.7–88.6)	7/42	–	20.7 (5.3–41.2)	–
Baker et al. [21]	Prospective (IV)	25	61.9 (39.6–81.8)	3/22	–	26.1 (13.8–41)	–
Abid et al. n [22]	Retrospective (IV)	20	40 ± 16 (16–60)	4/16	26 ± 4 (21–35)	44 ± 11 (26–65)	3 ± 2 (1–10)
Domínguez et al. [23]	Retrospective (IV)	23	51.34 ± 13.28	4/19	–	12	–
Coulomb et al. [24]	Retrospective (III)	17	53.5 ± 13.8 (17–71)	1/16	–	37.6 ± 10.4 (20–62)	2.9 ± 1.8 (0.5–9)
Wiese et al. [25]	Retrospective (III)	42 (45)	51 (17–61)	9/33	–	25 (12–48)	–
Govaert et al. [26]	Prospective (III)	5	–		–	6 weeks	–
Karlsson et al. [27]	Retrospective (IV)	33 (36)	43.2 ± 15.5	4/29	–	24.5 (24–100)	3.5 (1–21)
Annin et al. [28]	Prospective (III)	273	51.9 ± 12.5 (14.6–75.9)	37/236	28.1 ± 5.1(16.8–45.5)	44.4 ± 20.5 (24.1–109.2)	–
Thomassen et al. [29]	Retrospective (IV)	11	–	5/6	–	28 (15–42)	–

*LOE*: level of evidence; *FU:* follow up; –: not reported; *M:* male; *F:* female

### Diagnosis

In all studies reviewed, the diagnosis of GTPS was mainly based on clinical findings, supplemented by some radiological indicators and provocative injection tests.

The clinical findings were local tenderness over the GT area, 3 months of pain localized anterior or posterior to the greater trochanter refractory to conservative treatment. Positive single-leg stand and abductor strength were assessed, the latter by hip and knee extension with hip internal rotation and a resistant abduction test. Gait abnormalities, such as antalgic gait and/or Trendelenburg, were also noted.

Radiological evaluations were commonly performed to rule out concomitant hip or knee joint pathology. When involvement of the abductor tendon was suspected, plain radiographs of the affected hip were usually obtained, although they often did not yield significant findings except for possible calcifications at the insertion site of the tendon on the greater trochanter.

Ultrasound was used to evaluate abductor tendon thickening, tendinopathy, and the presence of partial or full-thickness tears. Calcific tendinopathy of the gluteal tendons could also be identified with ultrasound examination. Aspirations and injections were done under ultrasound guidance.

MRI was necessary when involvement of the gluteus medius and minimus tendons was suspected. MRI effectively identified partial and full-thickness tears, calcification of tendons, and muscle atrophy (fatty infiltration). Edema was recognized as the first MRI sign of gluteal tendinopathy. In addition, MRI can distinguish gluteal tendons from other tendons related to the greater trochanter, such as the piriformis syndrome, obturator internus, and obturator externus.

Only two studies used the Lall GTPS classification system [30] to categorize patients. Abid et al. [22] included patients classified as type I and II, while Annin et al.[28] included patients classified as type I, II and III.

### Surgical indications

In all studies, candidates for surgery for GTPS were those who presented with peritrochanteric pain or tenderness along with abductor weakness that persisted despite conservative measures. These measures typically included rest, non-steroidal anti-inflammatory drugs (NSAIDs), corticosteroid injections and physical therapy, administered for a minimum period of three months.

### Surgical technique

In nine out of ten studies (90%), the detailed treatment description received the maximum score of 10 points, while only Wiese et al. scored 5 points [25]. Patient positioning was predominantly lateral in all studies except Karlsson et al. and Annin et al., where a supine position was used [27, 28]. However, patient positioning was not specified in the study of Baker et al. [21].

In all included studies, patients underwent endoscopic trochanteric bursectomy, often in combination with iliotibial band or fascia lata release. In addition, endoscopic repair of gluteus medius (GM) tendon tears, trochanteric micro punctures to promote healing of the GM tendon, and suturing of the ITB to the greater trochanter (GT) in cases with coxa saltans have been performed. Some cases also involved endoscopic excision of calcifications, as shown in Table 2.

**Table 2:** Surgical position, surgical technique and postoperative therapy of patients following endoscopic treatment of GTPS

Authors (year)	Position	Surgical technique	Post op protocol
Drummond et al. [13]	lateral	Endoscopic bursectomy + vertical ITB release + PRP injection repaired endoscopically GM muscle tear (7 cases)	WB as tolerated with crutches for 6 weeks no formal postoperative rehabilitation program
Baker et al. [21]	-	Endoscopic bursectomy + long ITB incision	WB with crutches immediately physical therapy strengthening the hip and regain ROM
Abid et al. [22]	Lateral	Endoscopic bursectomy after cross-shaped incision in FL + in type II GTPS several micropunctures of apex of GT	WB with crutches recommended for 1 month. No rehabilitation prescribed
Domínguez et al. [23]	Lateral	Endoscopic bursectomy and ITB release	Assisted WB with two crutches and ROM first 2 weeks. Exercises on a static bicycle started at 1 week
Coulomb et al. [24]	Lateral	Endoscopic bursectomy and micro-perforations in the enthesis diamond-shaped ITB release (3 cases with snapping) calcification removal (2 cases)	unloading with two crutches for 6 weeks, then transverse deep fiber massage, active–passive ROM, and stretching exercises
Wiese et al. [25]	Lateral	Endoscopic bursectomy (4 cases with coxa saltans ITB sutured to the GT)	–
Govaert et al. [26]	Lateral	Endoscopic bursectomy + cross ITB incision	Full WB with 2 crutches during the first 2 weeks. After 8 weeks, patients can return to their sports activities
Karlsson et al. [27]	supine	Endoscopic bursectomy + FL lengthening FAI surgery (5 cases)	full ROM and WB hip and core strength and stability rehabilitation
Annin et al. [28]	supine	Endoscopic bursectomy ± trochanteric micropunctures ± GM tendon tear repair if found (189 cases)	Type I, II: WB with crutches + Hip brace first 2 weeks—rehabilitation from 2nd day. Type III: WB with crutches + Hip brace first 6 weeks—rehabilitation after 6 weeks
Thomassen et al. [29]	Lateral	Endoscopic star-shaped release of the ITB and bursectomy	Physiotherapy for stretching exercises and abductor training patients allowed full WB

*FL*: fascia lata; *PRP*: platelet rich plasma; *ITB*: iliotibial band; *FAI*: femoroacetabular impingement; *WB*: weight bearing; *ROM*: range of motion; *GM*: Gluteus Medius; *GT*: greater trochanter; –, not reported

Surgery time duration was reported in only three studies. Wiese et al. [25] reported an average operative time of 41 min (range 25–56), while Govaert et al. [26] documented 28 min on average (range 19–37) and Thomassen et al. [29] reported a mean of 30 min (range 19–40).

### Outcomes

Six studies used the Harris Hip Score (HHS) to report outcomes [21–24, 28, 29], while all studies except Thomassen et al. [29] included ratings on the Visual Analogue Scale (VAS). In addition, various other scoring systems were used, as shown in Table 3.

**Table 3:** Summary of post operative outcomes, complications, recurrences and revisions following endoscopic treatment of GTPS.

Authors and year	Pre-Operative outcomes measures; mean (range)	Post operative outcomes measures; mean (range)	Success rate	Recurrence	no of patients (complication)	Revision
Drummond et al. [13]	iHOT-33: 23.8 VAS: 7.8 OHS: 20.4	iHOT-33: 70.2 VAS: 2.8 OHS: 37.3	45/57 (78.9%)	7	0	0
Baker et al. [21]	VAS: 7.2 SF-36: 33.6 HHS: 51	VAS: 3.1 SF-36: 54 HHS: 77	(18/25) 72%	0	3 (pain, hematoma, seroma)	2 (1 drainage, 1 open)
Abid et al. [22]	VAS: 7 ± 1 (6–10) mHHS: 55 ± 9 (40–68) NAHS: 53 ± 6 (42–61)	VAS: 4 ± 2 (0–8) mHHS: 74 ± 12 (44–87) NAHS: 78 ± 15 (51–100)	100%	0	0	0
Domínguez et al. [23]	VAS: 8.1 WOMAC: 63.32 mHHS: 40.2 HOS Sport:18 HOS ADL:: 44.11	VAS: 0.48 WOMAC: 5.22 mHHS: 86.29 HOS Sport:: 77.9 HOS ADL: 89.77	100%	0	1 (neuroma)	0
Coulomb et al. [24]	VAS: 7.2 ± 1.1 (5–9) HHS: 53.5 ± 8.4 (36–68) Trendelenburg gait: 5 cases	VAS: 3.3 ± 1.9 (1–7) HHS: 79.8 ± 14.7 (45–96) Trendelenburg gait: 3 cases Satisfaction VAS: 6.2 ± 2.4 (0–9)	12/17 (70.6%)	1	4 (pain)	0
Wiese et al. [25]	VAS: 7.2 JOA disability score: 40.5	VAS: 3.8 JOA disability score: 72.6	44/45 (97.8%)	0	4 (hematoma)	0
Govaert et al. [26]	VAS: 75	VAS: 13	100%	0	1 (hematoma)	0
Karlsson et al. [27]	iHOT-12: 36.3 (14.5) VAS: 49.1 (20.9) EQ-VAS: 55.9 (17.3) HAGOS – quality of life: 23.4 (13.8) HAGOS – daily activity: 29.9 (28.0) HSAS: 1.74 (1.71)	iHOT-12: 54.0 (31.6) VAS: 58.5 (32.5) EQ-VAS: 63.3 (20.7) HAGOS – quality of life: 3.3 (30.8) HAGOS – daily activity: 53.1 (37.4) HSAS: 2.26 (1.48)	78%	1	1 (hematoma) 1 ( superficial infection)	1
Annin et al. [28]	mHHS: I(59.52 ± 15.4) II(57.27 ± 13.84) III(61.45 ± 12.18) NAHS:I(57.45 ± 17.35) II (61.39 ± 18.63) III (59.5 ± 12.48) iHOT-12: I(34.2 ± 19.11) II(30.38 ± 18.91) III(30.6 ± 13.16) HOS-SSS:I(31.73 ± 21.98) II(34.66 ± 27.83) III(37.92 ± 17.73) VAS: I(5.52 ± 2.33) II(5.39 ± 2.31) III(6.09 ± 1.85)	mHHS: I(83.3 ± 17.74) II(84.92 ± 16.22) III(85.83 ± 19.24) NAHS: I(83.31 ± 17.83) II(86.60 ± 14.51) III(83.33 ± 17.71) iHOT-12: I(74.58 ± 26.47) II(78.83 ± 21.92) III(79.38 ± 22.58) HOS-SSS: I(69.51 ± 28.8) II(76.25 ± 16.05) III(56.94 ± 31.48) VAS: I(2.35 ± 2.47) II(1.82 ± 2.12) III(1.99 ± 2.94)	7.9 /10	13	0	0
Thomassen et al. [29]	–	HHS: 73.81 (41–86) NRS pain: 4.3 NRS function: 3.1	10/11 (90.9%)	0	1 (pain)	0

*mHHS*: modified Harris hip score; *WOMAC*: Western Ontario and McMaster universities osteoarthritis index; *iHOT*-12: The international hip outcome tool; *HAGOS*: Copenhagen hip and groin outcome score; EQ-5D: EuroQoL-5 dimension questionnaire; *VAS*: Visual Analogue Scale; *NAHS*: non-arthritic hip score; *JOA*: Japanese orthopedic association; *iHOT*-12: international hip outcome tool; *HSAS*: hip sports activity scale; *HOS-SSS*: hip outcome score sport-specific subscale; *HOS ADL*: hip outcome score activities of daily living; *OHS*: Oxford hip score; *NRS*: numerical rating scale; – : not reported

A successful outcome was defined as complete pain relief or the absence of significant residual pain, as determined by the authors at final follow-up. Treatment success rates in the included studies ranged from 70.6–100%. Only the study by Govaert et al. reported the time to return to activity as 8 weeks [26].

### Complications

Several postoperative complications were reported in the included studies as shown in Table 3. Out of 22 patients with recurrence of symptoms only 2 patients underwent revision surgery, one of whom with open bursectomy at six months [21].

Additional complications included hematomas or seromas in 8 patients, with one postoperative seroma requiring surgical incision and drainage [21]. One study reported a cutaneous neuroma related to an endoscopic portal, which was successfully excised under local anesthesia three months after surgery [23]. Another study mentioned a single patient with a superficial wound infection after surgery, which resolved with antibiotic treatment [27].

## Discussion

The most important finding of this systematic review is that endoscopic hip surgery has been shown to be an effective surgical approach to relieve lateral hip pain associated with symptomatic external hip snapping (EHS) in patients with greater trochanteric pain syndrome (GTPS).

GTPS mainly affects very active young adults and it can significantly limit their activity levels. Concerning nonoperative treatment, Lievense et al. reported that the incidence of GPTS was 1.8 individuals per 1,000 per year. They found that after 1 year of symptoms, 36% of patients continued to experience discomfort, a percentage that stood at 29% after 5 years. In addition, the research indicated that 66% of patients treated with corticosteroids experienced complete improvement [1].

Although it is a common and debilitating condition, there is limited evidence on management options for GTPS. Most experts agree that the condition tends to resolve on its own. Corticosteroid injections and low-energy shock wave therapy (LESWT) are among the most studied therapeutic interventions. Surgery is generally considered only for patients who do not respond adequately to conservative treatments [31].

If conservative treatments fail to resolve the condition, surgical options, including open and endoscopic approaches, may be considered. Thomassen et al. reported endoscopic treatment for subjects who had shown poor response after at least one year of conservative treatment, which generally included stretching exercises, adductor training and possibly extracorporeal shock wave therapy (ESWT), as well as at least one steroid injection into the greater trochanter [29].

Most authors agree that GTPS is diagnosed primarily on the basis of clinical evaluation. However, the lack of clear diagnostic criteria and the resulting heterogeneity of studies make comparison difficult. Some authors have used imaging modalities to rule out other conditions [13, 22–28], while others have relied on a positive injection response as a diagnostic indicator [22–24, 27], and some have considered the absence of injection response as suggestive of GTPS resistant to conservative measures [21, 29]. In addition, some studies have focused on the management of medius gluteus tears, as these conditions often overlap, suggesting that excluding such tears with MRI may be helpful [26, 28].

Therefore, although clinical evaluation remains the gold standard for the diagnosis of GPTS, radiologic findings and provocation injection tests are often used to confirm the diagnosis.

Annin et al. evaluated abnormal gait, defined as antalgic and/or Trendelenburg gait, and assessed tenderness around the GT bilaterally. During the patient’s lateral positioning, adductor strength was assessed through the resisted abduction test, and diagnostic injections into the peritrochanteric space under ultrasound guidance were performed if necessary [28].

According to Coulomb et al., the diagnosis of GTPS was based on clinical findings such as lateral peritrochanteric pain and a positive Lequesne’s sign (pain during resisted external derotation with the hip flexed to 90 degrees). The diagnostic test consisted of evaluating the partial or complete reduction of pain following ultrasound-guided steroid injection into the trochanteric bursa. In addition, imaging findings, such as ultrasound or MRI, were used to detect peritrochanteric bursitis, tendinopathy of the gluteus medius or minimus, or any signs of chronic abductor tendon deficiency [24].

The duration between symptom onset and surgery seems consistent in recent years, with Karlsson et al. in 2023 [27] reporting 3.5 years, Abid et al. in 2022 [22] reporting 3 years, and Coulomb et al. 2016 reporting an average of 2.9 years. [24]*.*

Surgical interventions are generally reserved for cases refractory to conservative treatments. These surgeries may include bursectomy, iliotibial band (ITB) release, trochanteric reduction osteotomy, or gluteal tendon repair. Surgery often involves a combination of these approaches.

There is a consensus on the use of endoscopic techniques for the treatment of GTPS. All articles included in our review focused on endoscopic trochanteric bursectomy combined with ITB release or lengthening. Early evidence suggests that, when appropriately indicated, both trochanteric bursectomy and ITB release are effective in the management of GTPS. However, in Gluteus Medius enthesopathy (type II GTPS), some studies had described several micropunctures of the apex of the greater trochanter, along with repair of the gluteus medius (GM) tendon if torn [22, 28].

Although platelet-rich plasma (PRP) injection is commonly used in the management of other chronic tendinopathies, its efficacy for GTPS has not been widely explored. However, Drummond et al. conducted a study of 57 patients with GTPS treated endoscopically, also they injected PRP into the gluteal muscle–tendon junction after excision of the bursa, based on a theoretical premise to promote healing of gluteal tendinopathy with success rate of 80%. [13]

Although most of the studies report positive results, it is important to note that none of them included a control group. Due to variations in inclusion criteria and the limited number and heterogeneity of patients included, it is difficult to compare the results of different surgical methods.

In the past, GTPS was commonly addressed surgically through open incisions. However, after open procedures, reports indicated persistent hip pain in 6–31% of cases, recurrent snapping in 9–38% of cases, persistent hip flexor weakness in 3–42% of cases, and wound problems in 12–18% of cases[32].

In our review, we observed significantly an overall lower incidence rates of recurrence (4%) and revision (0.6%) after endoscopic treatment with GTPS, with minimal risk of complications. Importantly, most of the studies included in our review provided long-term follow-up data, improving the reliability of our results.

The study conducted by Baker et al. focused on arthroscopic bursectomy in a cohort of 25 patients, marking one of the first studies to assess outcomes using a validated, joint-specific scale, which is the Harris Hip Score. The results revealed satisfactory improvements in range of motion (ROM) and short- and medium-term function. In addition, the authors noted a significant decrease in pain levels, with scores on the visual analog scale (VAS) dropping from 7.2 to 3.1 over an average follow-up period of 26.1 months [21].

More recently, Karlsson et al. evaluated 33 patients and reported significant improvements in the primary outcome measure, the iHOT-12, following at least 2 years of follow-up after endoscopic treatment of GTPS. In addition, they observed that 71% of patients were satisfied with the procedure, along with a low incidence of complications [27].

This review identified a high rate of return to pre-injury activity levels. Dominguez et al. reported that all patients returned to their previous activities and only one patient experienced asymptomatic snapping at 1-year follow-up [23]. Evaluation of postoperative progress using the WOMAC scale demonstrated 100% improvement in pain and 91% improvement in snapping symptoms. These results were supported by Govaert et al. who similarly observed an improvement in quality of life, with all patients returning to sports activities within 8 weeks after surgery [26].

Although no serious complications were reported with the endoscopic procedure, the overall complication rate was 4% in this sample of 498 patients, with most complications consisting of hematomas or seromas. Therefore, in our opinion, the assumption that the endoscopic procedure is a "safe and easily reproducible procedure" could be justified.

The results of this systematic review, which includes multiple retrospective and prospective studies, provide a solid basis for future high-quality research. Endoscopic treatment of greater trochanteric pain syndrome (GTPS) has been shown to be a reliable and effective method of reducing pain and improving hip function.

This systematic review has some limitations that should be considered. First, the studies are subjected to various sources of bias in data collection and reporting, participant selection, and unblinded assessment of outcomes that could affect the validity and reliability of the study conclusions. Second, a wide variety of follow-ups with 1.6–65 months was reported in the different studies. A more homogeneous and standardized clinical and radiological follow-up could improve the data’s validity. Therefore, it is essential to interpret the results with caution and consider further research to confirm the results obtained in this systematic review.

Further research is needed to evaluate the long-term efficacy and cost-effectiveness of endoscopic treatment of GTPS. Large-scale prospective randomized studies with carefully selected control groups are essential to clarify the potential benefits of surgery for refractory GTPS. These studies should focus on refining surgical techniques and optimizing outcomes to provide clearer guidance to physicians and patients.

## Conclusion

This systematic review demonstrated that endoscopic surgery has emerged as a viable option for the management of GTPS that do not respond to conservative treatments, boasting a low risk of complications and a high likelihood of returning patients to pre-injury activity levels. Procedures such as endoscopic trochanteric bursectomy and iliotibial band release have been shown to be effective in relieving lateral hip pain, especially in cases of persistent GTPS. In addition, endoscopic GM tendon repair can be recommended in cases of partial or complete tears.

## Supplementary Information

Below is the link to the electronic supplementary material.

Supplementary file1 (XLSX 117 KB)

## Data Availability

Dataset analyzed in this study is available from the corresponding author on reasonable request.
